# An *in vivo* half-life extended prolactin receptor antagonist can prevent STAT5 phosphorylation

**DOI:** 10.1371/journal.pone.0215831

**Published:** 2019-05-07

**Authors:** Shengze Yu, Amira Alkharusi, Gunnar Norstedt, Torbjörn Gräslund

**Affiliations:** 1 Department of Protein Science, KTH Royal Institute of Technology, Stockholm, Sweden; 2 College of Medicine and Health Sciences, Sultan Qaboos University, Muscat, Oman; 3 Center for Molecular Medicine, Karolinska Institute, Solna, Stockholm, Sweden; National Research Council Canada, CANADA

## Abstract

Increasing evidence suggests that signaling through the prolactin/prolactin receptor axis is important for stimulation the growth of many cancers including glioblastoma multiforme, breast and ovarian carcinoma. Efficient inhibitors of signaling have previously been developed but their applicability as cancer drugs is limited by the short *in vivo* half-life. In this study, we show that a fusion protein, consisting of the prolactin receptor antagonist PrlRA and an albumin binding domain for half-life extension can be expressed as inclusion bodies in *Escherichia coli* and efficiently refolded and purified to homogeneity. The fusion protein was found to have strong affinity for the two intended targets: the prolactin receptor (K_D_ = 2.3±0.2 nM) and mouse serum albumin (K_D_ = 0.38±0.01 nM). Further investigation showed that it could efficiently prevent prolactin mediated phosphorylation of STAT5 at 100 nM concentration and above, similar to the PrlRA itself, suggesting a potential as drug for cancer therapy in the future. Complexion with HSA weakened the affinity for the receptor to 21±3 nM, however the ability to prevent phosphorylation of STAT5 was still prominent. Injection into rats showed a 100-fold higher concentration in blood after 24 h compared to PrlRA itself.

## Introduction

Prolactin (Prl) is a hormone that exerts its functions by homo-dimerization and activation of the prolactin receptor (PrlR) [1]. The Prl/PrlR axis is present in most vertebrates and is involved in more than 300 discrete biological functions, such as stimulation of body growth, stimulation of development during gestation, cell proliferation, homeostasis of different electrolytes etc. The major source of prolactin production in the human body is the pituitary gland. In addition, decidua, prostate, mammary and ovarian tissue as well as vascular endothelial cells and immune cells have been found to produce Prl locally [2]. The Prl/PrlR axis acts on several intracellular pathways [3]. One of the major signaling cascades involves activation of Janus kinase 2, which phosphorylates and activates several down-stream proteins including signal transducer and activator of transcription 5 (STAT5) [4]. Activated STAT5 forms a dimer that is translocated to the nucleus where it acts as a transcription factor on specific DNA elements.

We and others have previously found evidence to suggest that the Prl/PrlR axis can act to promote cancer development and would thus be a suitable target for development of cancer drugs. In glioblastoma multiforme (GBM), PrlR is often over-expressed, and over-expression is more common in patients with a more severe disease compared to patients with a less severe disease [5]. *In vitro*, blocking of Prl/PrlR signaling in the GBM cell line U251-MG was found to inhibit STAT5 phosphorylation and cellular invasiveness [5]. In another study, over-expression of PrlR in the GBM cell line G55, led to upregulated expression of Prl, which suggested that the upregulation of PrlR triggers an autocrine signaling loop [6]. This in turn was accompanied by an increased rate of proliferation. The increased rate of proliferation could be inhibited by addition of a specific inhibitor of Janus kinase 2, further showing the involvement of the Prl/PrlR axis in GBM cell proliferation. In ovarian cancer, a study showed that women with a family history of the disease had an elevated level of Prl [7]. In the same study it was found that exposure of normal ovarian epithelial cells to Prl eventually induced carcinogenesis, which suggested a link between elevated levels of Prl and hereditary ovarian cancer. Also, the PrlR receptor was often over-expressed in tumors from patients with ovarian cancer. Further *in vitro* studies have shown that the Prl/PrlR axis is active in some ovarian cancer cell lines to promote proliferation, cell migration and survival [8]. Studies on Prl and breast cancer have also revealed that a high circulating Prl level can be correlated to an increased risk of developing breast cancer, particularly in post-menopausal women [9,10].

The currently unmet treatment options for patients suffering from e. g. GBM, ovarian cancer and breast cancer, require development of novel modalities to be included in regimens for these diseases. Based on the above reports, an appealing approach is to investigate antagonists for Prl/PrlR mediated signaling.

**Fig 1:**
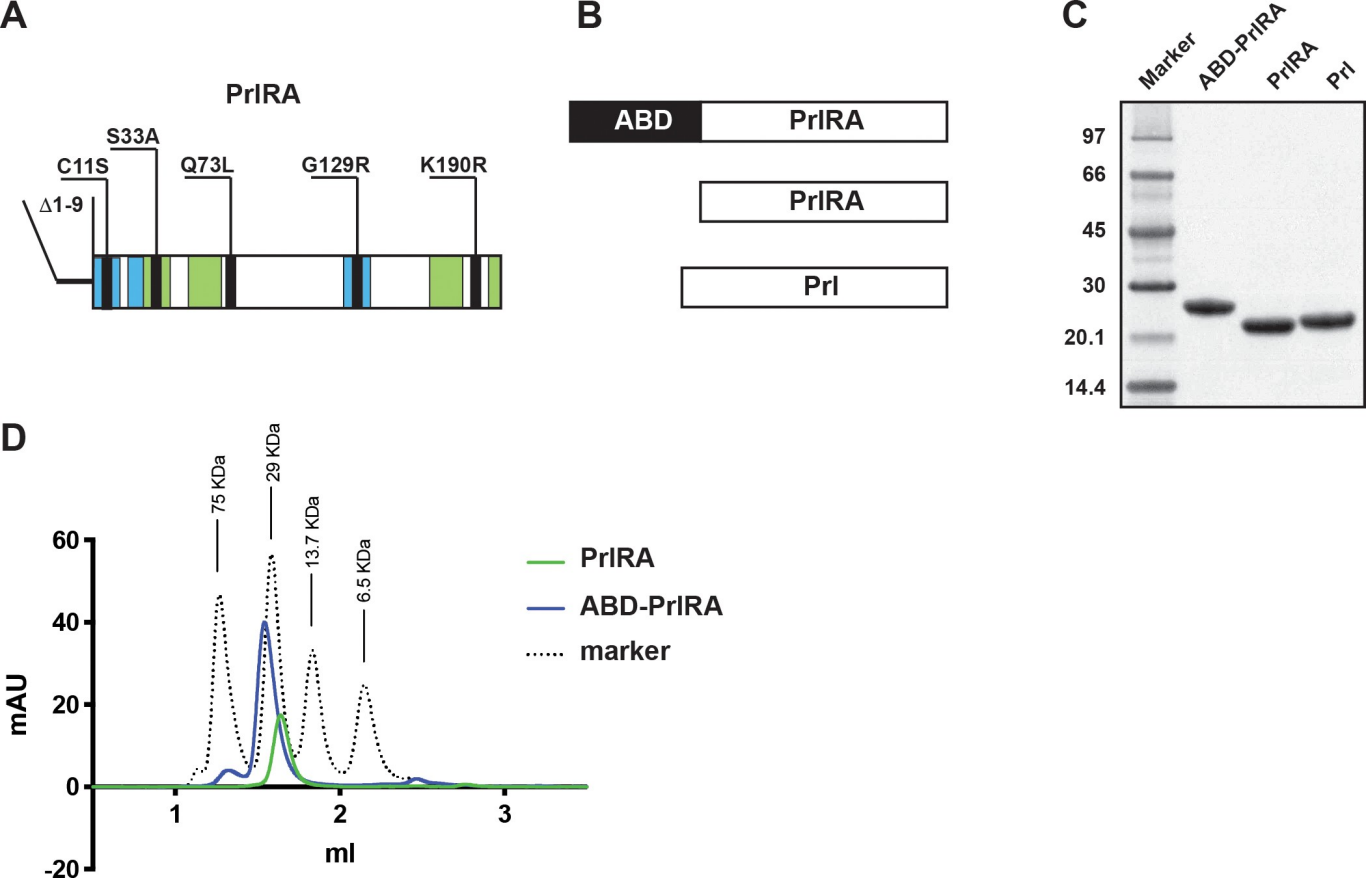
Construct design, production and purification. Panel A shows a schematic representation of PrlRA with depicted mutations. In green are the sections of PrlRA that composes binding site 1 and in blue are the sections that compose binding site 2. Panel B shows a schematic representation of the proteins investigated. After production and purification, the proteins were analyzed by SDS-PAGE on a 4–12% gradient gel under reducing conditions (Panel C, S1 Fig). Numbers to the left indicate the molecular weight of the marker proteins in kDa. Panel D shows an overlay of chromatograms recoded during size-exclusion chromatography analysis of PrlRA (green) and ABD-PrlRA (blue). Numbers above the marker peaks (black dotted) indicate the sizes of the marker proteins.

Prl has two sites of interaction with the PrlR and it appears that the hormone binds to preformed receptor dimers, leading to conformational changes and activation [11]. The binding sites in Prl are located on opposing sides of the hormone and one site has high affinity whereas the other has low affinity for the receptor. Previous efforts to develop a version of Prl that would act as an antagonist for PrlR have defined a variant with a G129R mutation (G129R-Prl) [12]. The rationale was to create the antagonist by mutations in the low affinity binding site, so that the antagonist would bind to one receptor molecule but prevent constructive interaction with a second receptor molecule. It would in turn prevent Prl-mediated activation of PrlR. The G129R-mutation considerably reduces the affinity of Prl for PrlR in the low affinity site. In most assays it was found to act as an antagonist, however in some assays G129R-Prl was found to still possess some agonistic activity [12], warranting development of more efficient antagonists. Despite this, G129R-Prl has been found to inhibit growth of breast and ovarian cancer xenografts in mice [13,14]. However, the procedure required daily administration or the use of slow releasing procedures to achieve a sufficient level in blood, due to the short half-life of the antagonist in circulation. It has previously been found that the greater the difference in affinity of the two binding sites on Prl for the PrlR, the more efficient antagonism can be achieved [15]. In a study by Bernichtein and co-workers, it was found that deleting the first nine amino acids in Prl led to an increased ability to activate the receptor; presumably by better stabilization of the dimer [16], and deletion of the same amino acids in G129R-Prl abolished the slightly agonistic property of the antagonist [17]. Further engineering efforts, focusing on increasing the affinity of the strong affinity site in Prl, resulted in identification of a variant with the amino acid substitutions: S33A, Q73L, G129R, K190R (Fig 1A). This variant had a 12-fold increase in affinity for PrlR and a 50-fold increase in antagonistic potency compared to G129R-Prl [18].

However, the molecular weight of Prl and the antagonistic variants described above is only 23 kDa, which is below the cut-off of approximately 60 kDa in the glomerular filter in the kidneys. It will therefore quickly be lost from circulation after injection. The measured half-life in circulation of Prl is only 41 min [19], which would likely be similar for the antagonists. The short half-life in circulation limits the applicability of the antagonists as cancer drugs and a version with longer half-life is desirable.

In order to investigate if a half-life extended prolactin receptor antagonist can be created, we designed and characterized a fusion protein consisting of an albumin binding domain and a prolactin receptor antagonist in this study. The albumin binding domain (ABD) was the G148-GA3 domain from *Streptococcal* protein G. This domain consists of 46 amino acids in a three-helix bundle structure [20]. It has previously been engineered for increased affinity by phage display-based selections from a library of gene-variants with point mutations, resulting in the variant ABD_035_ [21]. This variant was found to interact with serum albumin of human, cynomolgus, rat and mouse origin.

Once injected, this fusion protein may associate strongly with serum albumin in the blood, which should result in a complex that is larger than the glomerular filter in the kidneys and would thus be protected from filtration. In addition, association with serum albumin should also allow for interaction with the neonatal Fc receptor, which protects serum albumin and presumably the ‘piggybacking’ fusion protein from degradation by cells in contact with blood [22,23].

## Material and methods

### General

All chemicals were from Sigma Aldrich (Saint Louis, MO, USA) or Merck Millipore (Burlington, MA, USA) unless otherwise noted. Prolactin (Prl*) was obtained from Sino Biological (Beijing, China).

### Sub-cloning, expression and purification of the proteins

Genes encoding ABD-PrlRA, PrlRA and Prl were designed. Each gene started with the amino acid sequence MGSS. The ABD sequence used was the variant ABD_035_ [21]. The sequence encoding PrlRA was based on human prolactin with the following mutations: Δ1–9, C11S, S33A, Q73L, G129R, K190R [18] (Fig 1A). The genes encoding ABD-PrlRA, PrlRA and Prl were synthesized by Bio Basic (Markham, ON, Canada) and were delivered in the expression vector pET26 (Novagen, Darmstadt, Germany). Expression vectors were transformed to *Escherichia coli* BL21(DE3) cells (Novagen). Protein expression was carried out by growing the cells in 1L culture medium (Tryptic soy broth (30 g/l) with Yeast extract (5 g/l) (Merck Millipore) in a shake flask at 37°C. Expression was induced by addition of 1 mM Isopropyl-β-D-thiogalactopyranoside when OD_600_ was between 1.0 and 1.5 and was carried out for 3 h. The cells were harvested by centrifugation followed by resuspension of the cell pellet in lysis buffer (50 mM Tris, 5 mM EDTA, 0.1 M NaCl, 0.5% (v/v) Triton X-100, 5 mM 1,4-Dithiothreitol, pH 8.0). The cells were subsequently lysed by multiple passages through a French press (SLM instrument, Urbana, Il, USA.) and inclusion bodies (IBs) along with cell debris were collected by centrifugation. The pellets were washed twice with lysis buffer lacking Triton X-100 and were dissolved in denaturing buffer (100 mM Tris, 8 M Urea, 5 mM 1,4-Dithiothreitol, pH 8.0). Cell debris was pelleted by centrifugation and the supernatants were collected. The supernatants (approx. 40 ml) were diluted with an equal volume of refolding buffer (50 mM Tris, 1 mM EDTA, 0.1 M NaCl, 20% Glycerol, 1mM 1,4-dithiothreitol, pH 8.0) and proteins in the supernatants were refolded by dialysis against refolding buffer (2 l) for 1 d at 4°C, followed by dialysis against refolding buffer (2 l) lacking glycerol and 1,4-dithiothreitol. The supernatants were again diluted with an equal volume of water to a final volume of approx. 160 ml. Formed precipitates were pelleted by centrifugation and the cleared supernatants were filtered through a 0.45 μm Acrodisc Syringe Filter (PALL Life Science, Port Washington, NY, USA) and loaded on an 8 ml Q-Sepharose column (GE Healthcare, Chicago, IL, USA) that had been equilibrated with IEX running buffer. After extensive washing of the column with IEX running buffer, proteins were eluted with a NaCl gradient from 0 to 1 M. Column chromatography was carried out on an ÄKTA Explorer (GE Healthcare). Eluted material was pooled, followed by analysis by SDS-PAGE under reducing conditions, by separation on a 4–12% gradient NuPAGE gel (Thermo Fisher Scientific, Waltham, MA, USA). The gel was stained with GelCode Blue Stain Reagent (Thermo Fisher Scientific). Protein concentrations were determined using the Pierce BCA protein assay kit (Thermo Fisher Scientific) according to the supplier’s protocol. Size-exclusion chromatography analysis was carried out on an ÄktaPure FPLC system (GE Healthcare) using a Superdex 75 increase 5/150 GL column (GE Healthcare) and PBS as running buffer. The flow rate was 0.45 mL/min.

### Surface plasmon resonance

All interactions were measured on a Biacore 3000 (GE Healthcare Bio-Sciences, Uppsala, Sweden). PrlR (Sino Biological, Beijing, China), HSA and mouse serum albumin (MSA) (Sigma Aldrich) were immobilized on separate flow cells on CM5 chips at pH 4.6 in 10 mM sodium acetate buffer. Reference flow cells were created by activation and deactivation. All measurements were carried out at 25°C with a flow rate of 40 μl/min. The K_D_-values were calculated from the on- and the off-rates derived by BiaEvaluation software (GE Healthcare Bio-Sciences), using a 1:1 Langmuir interaction model.

To determine the equilibrium dissociation constants (K_D_) of Prl, Prl*, PrlRA and ABD-PrlRA for the PrlR, dilution series of each purified protein were injected over the chip surface with immobilized PrlR. Each dilution series was injected in four independent analyses, except for Prl which was injected in two independent analyses. PBS supplemented with 0.05% Tween-20 was used as running buffer and for dilution of the analytes. Regeneration of the chip surface was carried out by injection of 50 mM Sodium Acetate (pH 5.0). For determination of the interaction between the SA/ABD-PrlRA complex and PrlR, HSA was added to a dilution series of ABD-PrlRA to a final concentration of 400 nM in each sample, followed by incubation for 4 h at 4° C. The dilution series was subsequently injected over a chip surface with immobilized extracellular domain of PrlR. The dilution series was injected in four independent analyses. PBS supplemented with 0.05% Tween-20 and 400 mM HSA was used as running buffer and for dilution of the analytes. Regeneration of the chip surface was carried out by injection of 50 mM Sodium Acetate (pH 5.0).

**Fig 2:**
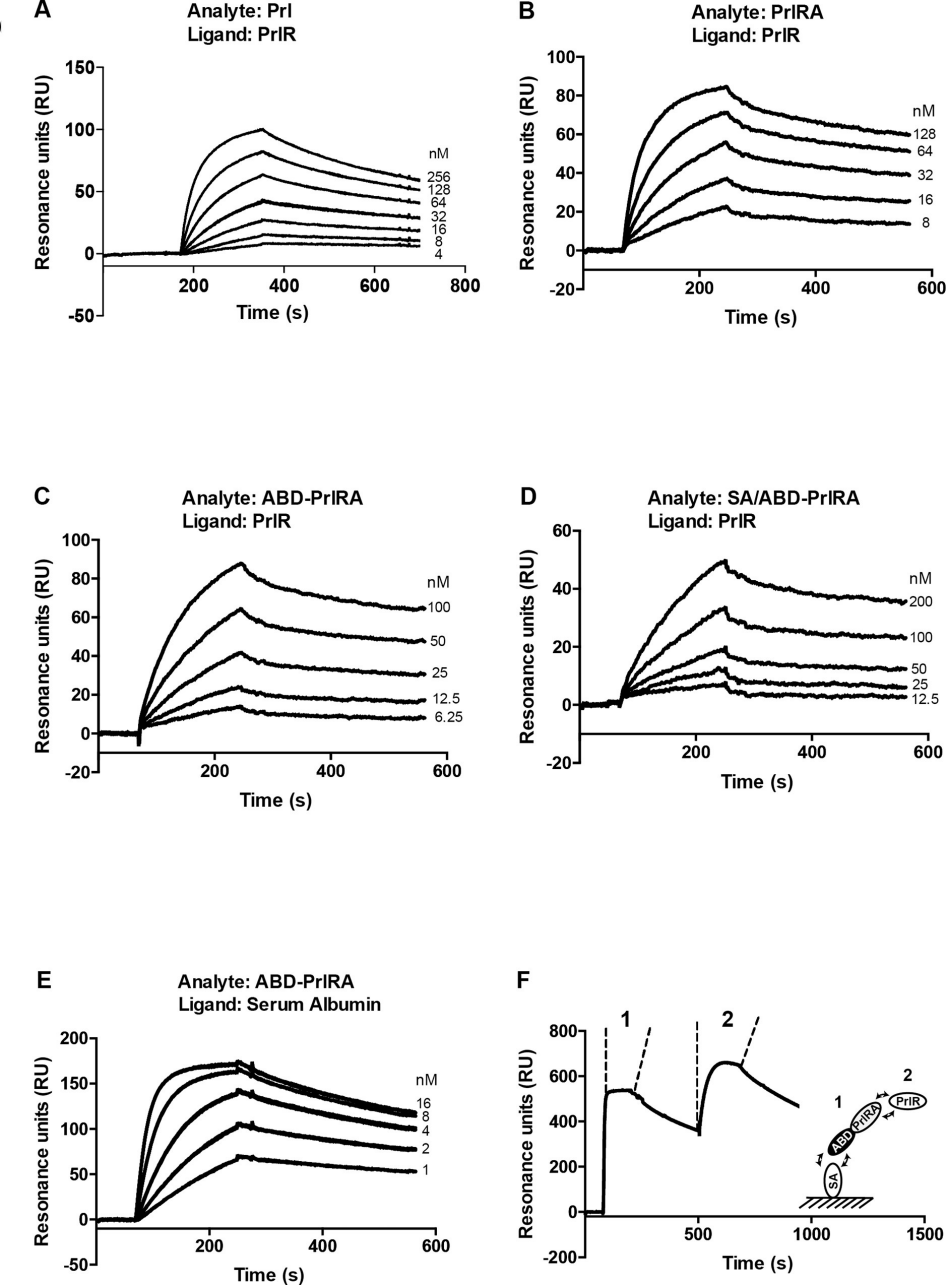
Affinity measurement by real-time biosensor analysis. Panel A-D shows overlays of representative sensorgrams obtained after injection of dilution series of ABD-PrlRA, PrlRA, Prl and SA/ABD-PrlRA (analyte) from low to high concentration over a flow-cell with immobilized PrlR (ligand). The surface density of PrlR was approximately 300 RU. Numbers to the right of the panels correspond to the concentration span of the dilution series. Panel E shows an overlay of representative sensorgrams after injection of a dilution series of ABD-PrlRA over a flow cell with immobilized mouse serum albumin. The surface density of MSA was approximately 800 RU. Numbers to the right correspond to the span of concentrations of the dilution series. (Panel F) A co-binding experiment was performed where ABD-PrlRA (50 nM) was injected over a flow cell with immobilized mouse serum albumin (1) followed by injection of PrlR (100 nM)(2). The panel shows the recorded sensorgram and the figure to the right is a graphical depiction of the experiment.

In the co-binding experiment (Fig 2E), HBS-EP buffer (10mM HEPES, 150mM NaCl, 3.4mM EDTA, 0.05% Tween 20, pH 7.4) was used as running buffers and for sample dilution.

To determine the affinity of the ABD-PrlRA/HSA interaction, a dilution series of ABD-PrlRA was injected over a surface with immobilized HSA. The dilution series was injected in two independent analyses. HBS-EP buffer was used as running buffers and for sample dilution. The surface was regenerated by injection of 15 mM HCl.

### Cell culture

The glioblastoma cells line (U251-MG) was obtained from American Tissue Culture Collection (ATCC, Manassas, VA, USA) and was cultured in Dulbecco’s Modified Eagle’s Medium supplemented with 10% fetal bovine serum, non-essential amino acids, 1% sodium pyruvate and 100 U/ml penicillin in 5% CO_2_ atmosphere at 37°C. All chemicals were from Invitrogen (Carlsbad, CA, USA) except for non-essential amino acids, which was from Sigma Aldrich.

### STAT5 phosphorylation assay

The level of phosphorylation of STAT5 in U251-MG was determined as follows: cells were seeded in 6-well plates (700 000 cells/well) and were allowed to attach for 24 h in complete medium. The cells were then starved in serum free medium over-night. Dilution series of ABD-PrlRA and PrlRA, in serum free medium supplemented with 400 nM human serum albumin, were prepared and added to the cells followed by incubation for 30 min. Prl (100 ng/ml) was added followed by incubation for 20 min to induce phosphorylation of STAT5. The cells were washed twice with ice cold phosphate buffered saline (PBS; Gibco’s PBS Tablets, Thermo Fisher Scientific) and lysed by adding 250 μl ice-cold cell lysis buffer (20 mM Tris, 137 mM NaCl, 10% glycerol, 2 mM EDTA, 1% (v/v) NP-40, 1 mM activated sodium orthovanadate, complete EDTA-free protease inhibitor cocktail (Roche, Mannheim, Germany), pH 8.0). Cells were detached with a cell scarper, mixed thoroughly in the cell lysis buffer with a pipet and were stored at -80°C until analyzed.

On the day of analysis, the cell samples were subjected to 3 freeze/thaw cycles (37°C/-80°C) to release the intracellular fraction followed by centrifugation to obtain a clear supernatant. The protein concentrations of the supernatants were determined using the Pierce BCA Protein Assay Kit (Thermo Fisher Scientific), essentially according to the manufacturer’s protocol. Samples, each containing 25 μg protein, of the supernatants were separated by SDS-PAGE under reducing conditions on a 4–12% gradient NuPAGE gel. Separated samples were blotted onto a polyvinylidenediflouride membrane (Invitrogen), followed by over-night blocking of the membrane with 5% bovine serum albumin in Tris-Buffered Saline (20 mM Tris, 150 mM NaCl, pH 8.0) supplemented with 0.1% (v/v) Tween-20. The membrane was first incubated with P-STAT5(Y694) Rabbit antibody (Cell Signaling, Danvers, MA, USA) for 90 min at r.t. to detect phosphorylated STAT5. Membranes were visualized by incubations with an appropriate HRP-conjugated anti-Rabbit IgG secondary antibody (Cell Signaling) and were developed with the ECL Western blotting detection system (Millipore) according to the manufacturer's protocol. After determination of STAT5 phosphorylation, the membrane was stripped by incubation with stripping buffer (0.15% Glycine, 0.1% SDS, 1% Tween-20, pH 2.2) for 7 min at r.t., followed by reblotting with an anti-STAT5 Rabbit antibody (Cell Signaling) and the same secondary antibody as above. Membranes were developed by same method as above to detect total STAT5.

### Pharmacokinetic study

Male Wistar rats, 12 weeks old, were obtained from Harlan Laboratories (Horst, Netherlands) and were used to investigate the pharmacokinetic properties of ABD-PrlRA and PrlRA. The experiment was planned and performed in accordance with national legislation on protection of laboratory animals. The animal study was approved by Ethical committee in Stockholm, South (Stockholms södra djurförsöksetiska nämnd, Ethical Permit S7-15), Sweden. Animals were kept under controlled temperature and light conditions. Each cage contained three animals approximately 6 month of age that were given free access to water and food. Following acclimatization to human handling, a single dose of each protein (4 mg/kg) in phosphate buffered saline was subcutaneously injected. Blood samples (less than 300 μl) were collected from the tail vein 24 h post-injection following euthanasia by exposure to carbon dioxide and were used to prepare serum. A group of untreated animals served as control. Serum levels of human prolactin was measured using the Elecsys Prolactin II test in a Cobas instrument (Roche) according to the manufacturer’s protocol. Briefly, two different antibodies against Prl is used in a sandwich setup, where the first is used to capture the hormone onto an electrode via a streptavidin/biotin interaction and the second is Ruthenylated and used for detection by a chemiluminescent assay after applying voltage to the electrode.

### Statistical analysis

Statistical analysis was done using Prism 8 (Graphpad, La Jolla, CA, USA). A one-way ANOVA with Tukey’s post hoc multi-comparisons test of the equilibrium dissociation constants was performed. Each group consisted of values from four independent experiments except for the Prl group which consisted of values from two independent experiments. P<0.05 was considered to be the cut-off for statistical significance.

## Results

### Construct design, production and purification

To create a potentially half-life extended prolactin receptor antagonist (PrlRA), a fusion protein was designed which consisted of PrlRA with an N-terminal ABD extension, ABD-PrlRA (Fig 1B). The protein was produced intracellularly as inclusion bodies in *Escherichia coli* under control of the strong T7-promoter at 37°C. For use as controls, PrlRA without ABD extension and prolactin (Prl) were similarly expressed. Following washing and solubilization of the inclusion bodies, the proteins were refolded and were purified by anion exchange chromatography. Eluted proteins were pooled and analyzed by SDS-PAGE, showing pure proteins of essentially the expected molecular weight (Fig 1C). Samples of PrlRA and ABD-PrlRA were analyzed by size exclusion chromatography under native conditions and were essentially eluted as single symmetrical peaks with an elution volume corresponding to the size to a monomer (Fig 1D), indicating a homogenous and correctly folded product. The final yield for Prl, PrlRA and ABD-PrlRA was 0.7, 1.7 and 2.5 mg/L cell culture, respectively.

### Detailed investigation of the affinities

To study the interaction of Prl, PrlRA and ABD-PrlRA with the prolactin receptor (PrlR), a real-time biosensor analysis was performed. As positive control Prl obtained from a commercial source was included (Prl*). Dilution series of ABD-PrlRA, PrlRA and Prl were sequentially injected from low to high concentration over a surface with immobilized PrlR (Fig 2A–2C). To mimic an *in vivo* environment, the complex of SA and ABD-PrlRA (SA/PrlRA) was formed and a dilution series was similarly injected from low to high concentration (Fig 2D). The kinetic constants were derived assuming a 1:1 Langmuir interaction and are presented in Table 1. The equilibrium dissociation constants (K_D_-values) for ABD-PrlRA, PrlRA, Prl and SA/ABD-PrlRA were determined from the kinetic constants to 2.3±0.2, 3.4±0.5, 8.4±0.2 and 21±3 nM, respectively. The affinity of Prl* was similarly determined to 23±4 nM (S2 Fig). The results show no statistically significant difference in the affinities of PrlRA and ABD-PrlRA for PrlR suggesting that an N-terminal extension of PrlRA with the ABD does not interfere with the ability to interact with the PrlR. Neither PrlRA nor ABD-PrlRA had a significantly different affinity for the PrlR compared to in house produced Prl. However, both PrlRA and ABD-PrlRA had significantly stronger affinity (P<0.0001) for PrlR compared to the commercially obtained Prl*. The complex between SA/ABD-PrlRA had an affinity similar to Prl* which is a significant (P<0.0001), 10-fold reduction, compared to the affinity of ABD-PrlRA for the PrlR. The affinity of ABD-PrlRA for serum albumin (SA) from mouse was similarly determined and was found to be 0.38±0.01 nM (Fig 2E, Table 2). A strong affinity for SA is indicative of a potentially prolonged half-life *in vivo*. To investigate if ABD-PrlRA was able to simultaneously interact with PrlR and SA, a co-binding experiment was performed (Fig 2F). ABD-PrlRA was first injected over a flow cell with immobilized SA to establish the ABD/SA interaction. During the dissociation phase, PrlR was injected over the surface and a second increase in the biosensor signal was recorded, which strongly suggests that a tripartite complex consisting of SA, ABD-PrlRA and PrlR was formed. As control, PrlR was directly injected over the flow cell with immobilized SA, which gave no increase in biosensor signal (S3 Fig).

**Table 1:** Kinetic constants for interaction with PrlR.

	ABD-PrlRA	PrlRA	Prl	SA/ABD-PrlRA
k_a_ (1/Ms)	3.3±0.3×10^5^	2.6±0.6×10^5^	1.4±0.1×10^5^	3.9±0.8×10^4^
k_d_ (1/s)	7.4±0.1×10^−4^	8.8±0.3×10^−4^	1.2±0.1×10^−3^	7.8±0.3×10^−4^
K_D_ (nM)	2.3±0.2	3.4±0.5	8.4±0.2	21±3

**Table 2:** Kinetic constants for interaction between ABD-PrlRA and SA.

	ABD-PrlRA
k_a_ (1/Ms)	3.1±0.1×10^6^
k_d_ (1/s)	1.2±0.1×10^−3^
K_D_ (nM)	0.38±0.01

### Blocking of STAT5 phosphorylation

**Fig 3:**
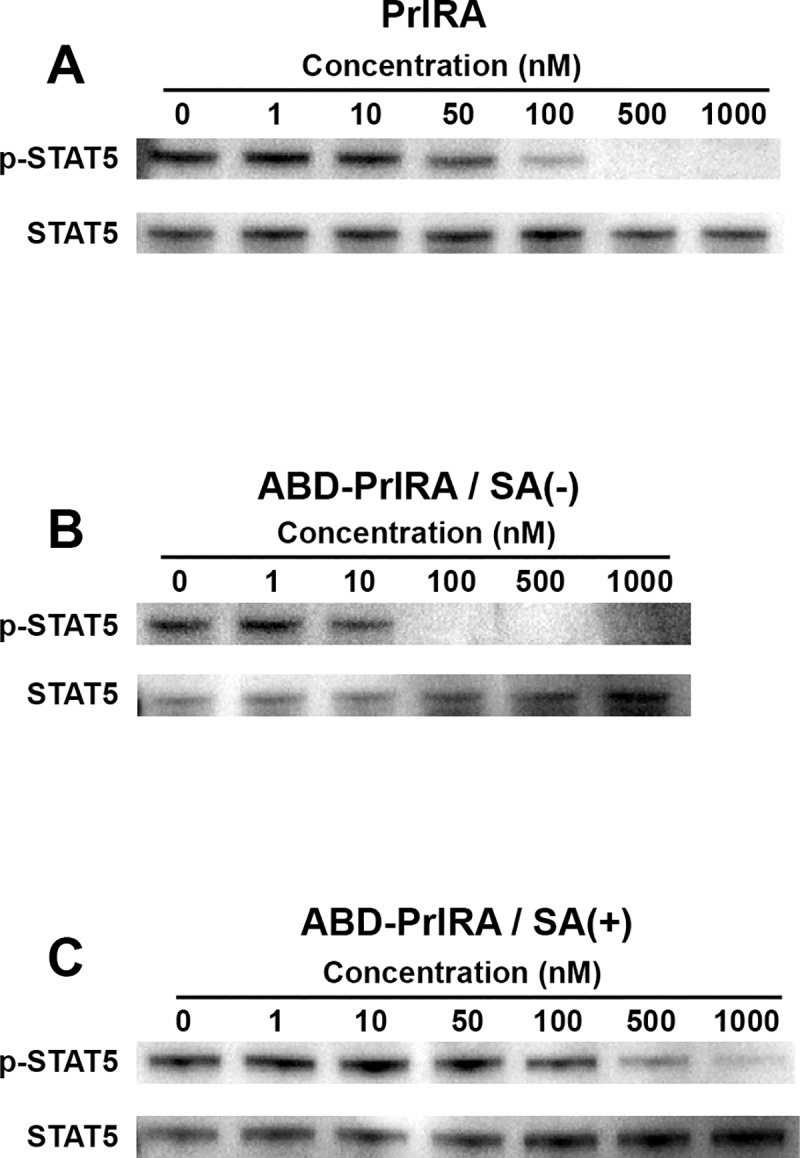
Effect of PrlRA and ABD-PrlRA on Prl-mediated STAT5 phosphorylation in U251-MG cells. The cells were seeded in 6-well plates and were starved over-night under serum free conditions. On the following morning, the cells were treated with different concentrations of PrlRA or ABD-PrlRA in the presence or absence of SA, followed by induction of STAT5 by Prl for 20 min. The level of phosphorylated STAT5 was determined by analysis of the intracellular content in each well by SDS-PAGE, followed by Western blotting using an antibody recognizing phosphorylated STAT5 (upper part of each panel). The membrane was then stripped and probed with an antibody recognizing both phosphorylated and non-phosphorylated STAT5 (lower part of each panel). Panel A shows the results using a dilution series of PrlRA, panel B shows the results using a dilution series of ABD-PrlRA in the absence of SA, panel C shows the results using a dilution series of the ABD-PrlRA/SA complex. The numbers above each lane indicates the concentration of PrlRA or ABD-PrlRA added.

To investigate the possible antagonistic effect of ABD-PrlRA and PrlRA on PrlR signaling, a STAT5 phosphorylation assay was performed on U251-MG cells. It has previously been shown that prominent STAT5 phosphorylation can be induced in this cell line by addition of 100 nM Prl to the culture medium [5]. The cells were treated with different concentrations of PrlRA or ABD-PrlRA in the presence or absence of SA for 30 min to potentially block PrlR on the cells, prior to induction of STAT5 phosphorylation by addition of Prl. SA was added to emulate an *in vivo* situation and the concentration used, 400 nM, theoretically results in that essentially all ABD-PrlRA is in complex with SA, based on the determined equilibrium dissociation constant (Table 2). The results showed that Prl can induce phosphorylation of STAT5 in the absence of PrlRA (Fig 3, lane 0 nM in all panels). However an addition of 100 nM PrlRA reduces phosphorylation and at 500 nM and above, phosphorylation is completely abolished (Fig 3A). Addition of ABD-PrlRA at a concentration of 100 nM or above completely abolished phosphorylation of STAT5 (Fig 3B). Addition of the complex consisting of ABD-PrlRA and SA resulted in reduction of STAT5 phosphorylation in a dose dependent manner at 500 nM and 1000 nM.

### ABD prolongs the *in vivo* half-life of the PrlRA

**Fig 4:**
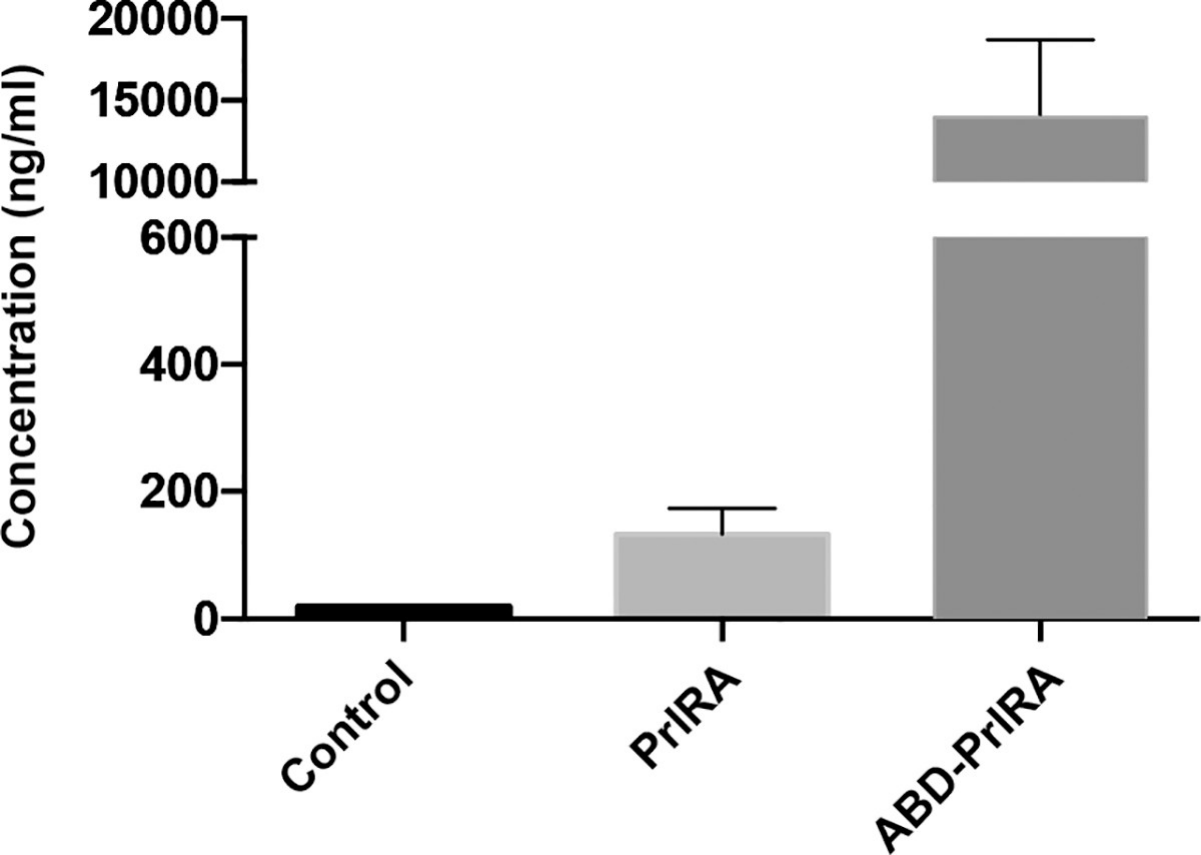
*In vivo* half-life extension of PrlRA. The PrlRA and ABD-PrlR were s.c. injected (4 mg/kg) in Wistar rats (n = 4). 24 h after injection, blood was collected and the concentration of PrlRA and ABD-PrlRA was determined.

To examine the pharmacokinetic performance of PrlRA and the potentially half-life extended ABD-PrlRA, both proteins were subcutaneously given to Wistar rats at a concentration of 4 mg/kg. Since mouse and rat serum albumin share a high sequence identity and since ABD-PrlRA could interact strongly with mouse serum albumin, it was expected that it could likewise interact with rat serum albumin. 24 h after injection, blood samples were collected and the concentration of ABD-PrlRA and PrlRA were determined (Fig 4). The results showed that the serum concentration of PrlRA was 150 ng/ml, while the concentration of ABD-PrlRA was 15.000 ng/ml (100 fold higher), showing that the addition of ABD increased the *in vivo* half-life of PrlRA. A group of untreated animals (control) were used to ensure that that the assay to detect human Prl in serum did not detect rat Prl.

## Discussion

Long acting antagonists of signaling through the Prl/PrlRA axis are highly attractive due to their potential as drugs to treat a number of different diseases, including cancer of various kinds. In this study, we have designed and investigated a half-life extended prolactin receptor antagonist (PrlRA) in the form of a fusion protein consisting of the antagonist with an N-terminal albumin binding domain (ABD). Since the proposed mechanism of action of the PrlRA is based on its strong interaction with only one prolactin receptor molecule, an important consideration was where to attach the ABD so that it would not interfere with the PrlRA/PrlR interaction. The ABD has been found to endow fusion proteins with an extended half-life when placed either at the N- or C-terminus or in the middle of a fusion protein [24–26], so the placement would likely not be constrained by the ABD. The crystal structure between N-terminally truncated G129R-Prl and the PrlR has been determined [27] and clearly shows that the C-terminus of G129R-Prl is directly engaged in the interaction with PrlR, but the N-terminus is more distant from the interface. Based on the crystal structure, the ABD was therefore placed in the N-terminus of the PrlRA.

The equilibrium dissociation constants (K_D_) of the interactions between ABD-PrlRA or PrlRA and the PrlR was similar. In the original publication on identification of the PrlRA [18] the equilibrium dissociation constant was determined to 0.9 nM for the PrlRA/PrlR interaction which is slightly stronger than the affinities measured in the present study. However, even though not significantly different, the affinity of both PrlRA and ABD-PrlRA was stronger than the affinity of Prl for PrlR, which is in line with the publication by Liu *et al*. [18]. The affinity of the SA/ABD-PrlRA complex for the PrlR was weaker than the affinity of ABD-PrlRA alone. This shows that even though the ABD-fusion does not affect the affinity for the PrlR, association with SA does, and is likely a consequence of sterical hindrance since SA is a relatively large protein compared to ABD-PrlRA. The measured kinetic constants of the SA/ABD-PrlRA complex and the PrlR is also an approximation since a 1:1 Langmuir interaction is assumed, even though there are two simultaneous interactions taking place; the SA/ABD-PrlRA interaction and the ABD-PrlRA/PrlR interaction. During the course of the biosensor analysis a slow inactivation (approximately 15–20%) of the surface took place. It does not affect the measured affinity constants but affects the absolute responses obtained. The absolute responses for the SA/ABD-PrlRA (Fig 2D) was expected to be higher compared to the responses obtained for ABD-PrlRA alone (Fig 2C). The above factors together with the weaker affinity is likely responsible for the lower response levels obtained.

The affinity (K_D_) of ABD-PrlRA for serum albumin was 0.4 nM, which should lead to an increase in the *in* vivo half-life. Indeed, a remarkable difference (100 fold) in concentration in blood was observed 24 h after injection in rats. Previous studies of the interaction between ABD and serum albumins from different species have shown that the interaction with human serum albumin is much stronger than the interaction with rat and mouse serum albumin [21]. The *in vivo* half-life of human serum albumin is also longer than the *in vivo* half-life in rats which suggests that the half-life extension should be at least as efficient in humans. We also found that ABD-PrlRA could bind to serum albumin and the PrlR simultaneously. It is an important finding that ABD-PrlRA can interact with the PrlR while bound to serum albumin, since it will be constantly engaged by serum albumin *in vivo*, due to the strong affinity and high concentration of serum albumin in blood. The formation of the tripartite SA/ABD-PrlRA/PrlR complex in SPR strongly suggests that ABD-PrlRA will be able to bind to and inhibit signaling though the PrlR also *in vivo*.

The ABD was used in this study to increase retention in circulation. Even though the actual half-life was not quantified, it is obvious from the marked increase in blood concentration after 24 h, that addition of the ABD increased the half-life. Typically, addition of an ABD extends the half-life through interaction with albumin [28], but other factors, such as higher stability in serum conferred by genetic fusion to the ABD, cannot be ruled out. There are alternatives to using an ABD for half-life extension [29], e.g. by addition of polyethylene glycol (PEG) which increases the stokes radius. In an investigation on human growth hormone, a hormone related to prolactin in size, structure and function, 5 kDa PEG polymers were attached to primary amines using unspecific chemistry, until a version with increased serum half-life was obtained [30]. However, each added PEG polymer decreased the activity and increased EC_50_ in a cell-based assay, which suggests that PEGylation is likely a suboptimal half-life extension procedure for the PrlRA. Other possibilities include direct fusion with serum albumin [31] or fusion with the Fc part or IgG [32]. Both strategies are extensively used for *in vivo* half-life extension of many different proteins and peptides but typically require more advanced hosts than *E*. *coli* for production, which increases the cost-of-goods. Fc-fusion also generally results in a homodimeric construct which may not always be desired, even though strategies to create monomeric Fc-fusions have been described [33].

In the inhibition-of-STAT5-phosphorylation assay, ABD-PrlRA and PrlRA were equally potent inhibitors of the PrlR, and this is in line with the finding that they have the same affinity for the PrlR. However, inhibition of STAT5 phosphorylation by ABD-PrlRA was reduced in the presence of serum albumin, indicating that the complex can be formed but that serum albumin hinders binding to the PrlR. This is likely a consequence of the weaker affinity of the SA/ABD-PrlRA for the PrlR. However, the affinity was still strong (K_D_ 23±3 nM) and prominent inhibition of STAT5 phosphorylation was observed. Reduced efficiency of ABD containing fusion proteins in the presence of serum albumin has previously been reported, e.g. for a single-chain diabody [34].

In summary, an ABD-PrlRA fusion protein was investigated and both domains were found to be functional *in vitro*. In rats, the fusion protein showed an enhanced pharmacokinetic performance.

## Supporting information

S1 Fig Analysis of purified proteins by SDS-PAGE. Analysis was carried out on a 4–12% gradient gel under reducing conditions. From left to right: Molecular weight marker, ABD-PrlRA, PrlRA, Prl.
(TIF)

S2 Fig Affinity measurement by real-time biosensor analysis. Dilution series of commercially obtained Prl* were sequentially injected from low to high concentration in four independent experiments over a flow-cell with immobilized PrlR (ligand). The panel shows an overlay of representative sensorgrams recoded after injection of two of the dilution series. The on- and the off-rates were derived by BiaEvaluation software (GE Healthcare Bio-Sciences) using a 1:1 Langmuir interaction model. The equilibrium dissociation constant was determined from the on- and off-rate to 23±4 nM.
(TIF)

S3 Fig Analysis of interaction between PrlR and SA. 100nM PrlR was injected with a flowrate 40ul/min over the SA surface from 60 to 180 s in the figure. As expected no response was detected.
(TIF)
