# Supplementary figures and images for: An in vivo half-life extended prolactin receptor antagonist can prevent STAT5 phosphorylation
Source: PLoS One. 2019 May 7;14(5):e0215831. doi: 10.1371/journal.pone.0215831 (PMC6504076; doi:10.1371/journal.pone.0215831)

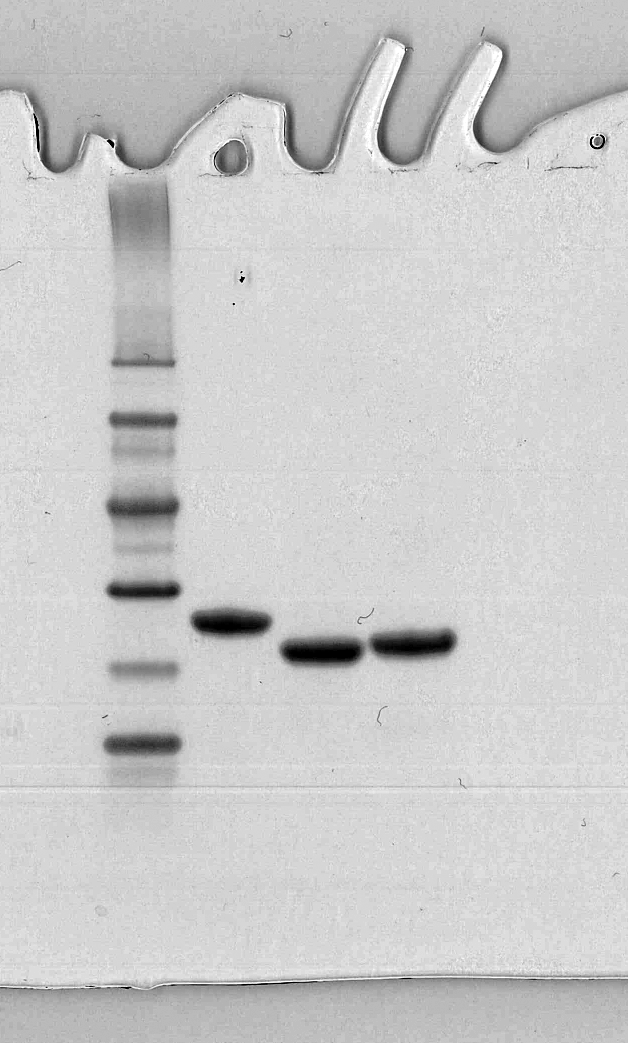

Supplement: S1 Fig — Analysis was carried out on a 4–12% gradient gel under reducing conditions. From left to right: Molecular weight marker, ABD-PrlRA, PrlRA, Prl. (TIF) [file pone.0215831.s001.tif]

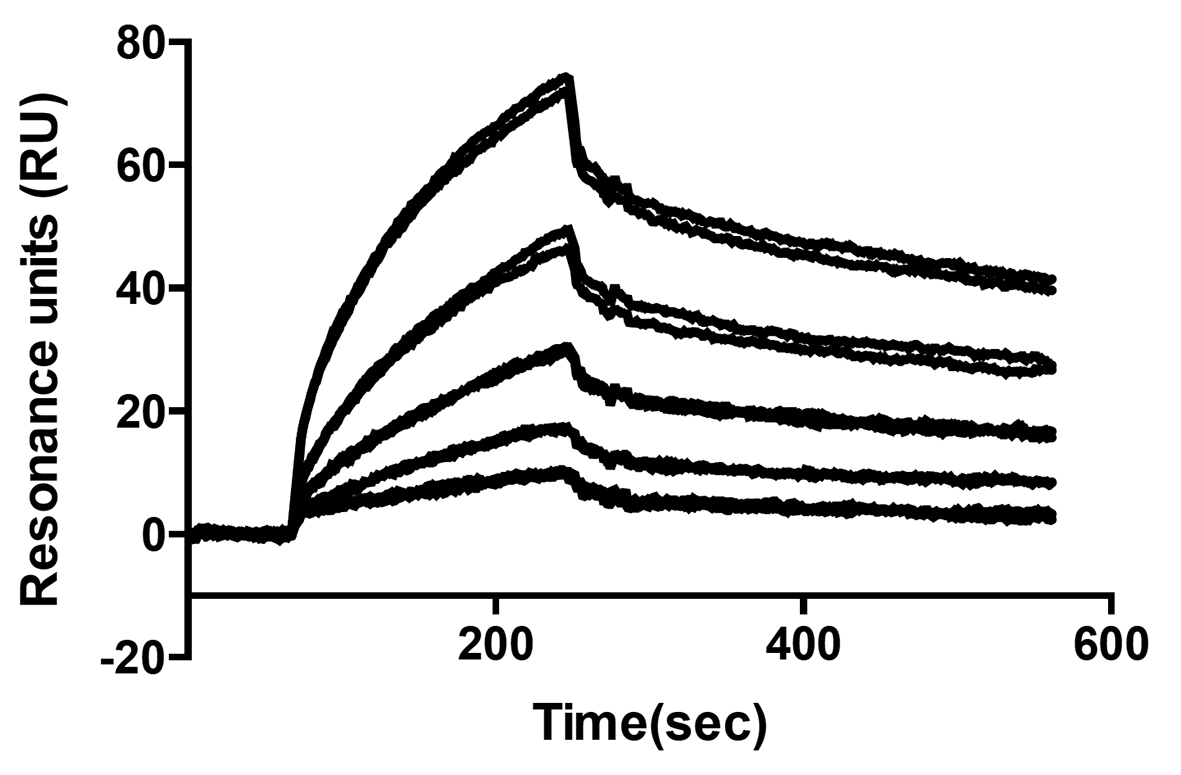

Supplement: S2 Fig — Dilution series of commercially obtained Prl* were sequentially injected from low to high concentration in four independent experiments over a flow-cell with immobilized PrlR (ligand). The panel shows an overlay of representative sensorgrams recoded after injection of two of the dilution series. The on- and the off-rates were derived by BiaEvaluation software (GE Healthcare Bio-Sciences) using a 1:1 Langmuir interaction model. The equilibrium dissociation constant was determined from the on- and off-rate to 23±4 nM. (TIF) [file pone.0215831.s002.tif]

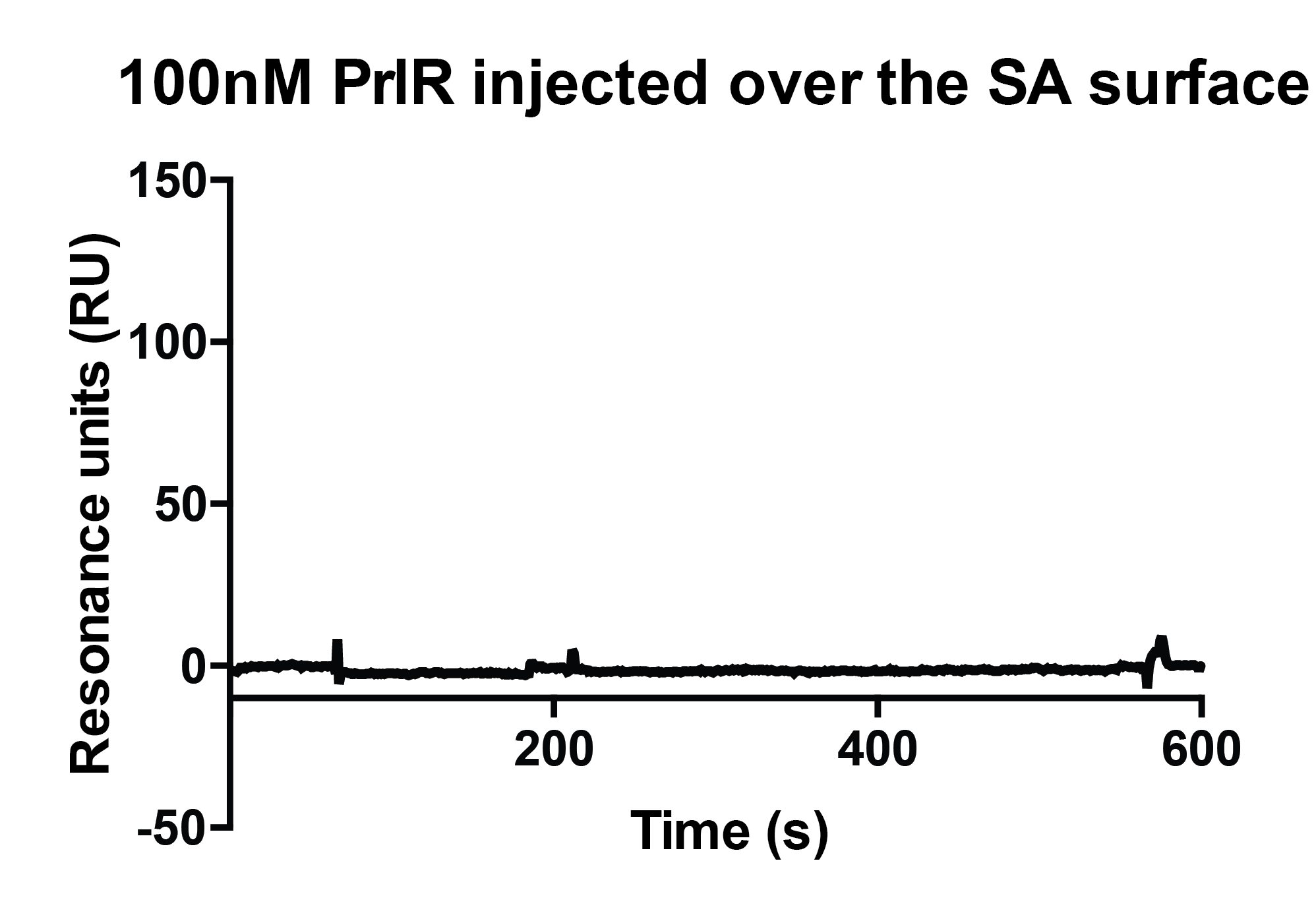

Supplement: S3 Fig — 100nM PrlR was injected with a flowrate 40ul/min over the SA surface from 60 to 180 s in the figure. As expected no response was detected. (TIF) [file pone.0215831.s003.tif]
